# An expert patient program to improve the empowerment and quality of life of people with multiple sclerosis: protocol for a multicenter pre-post intervention study

**DOI:** 10.3389/fneur.2023.1172640

**Published:** 2023-05-18

**Authors:** Miguel Angel Robles-Sanchez, Paloma Amil-Bujan, Cristina Bosch-Farré, Clàudia Coll-Martínez, Maria Jesús Arévalo, Elisenda Anglada, Rebeca Menéndez, Xavier Montalban, Jaume Sastre-Garriga, Lluís Ramió-Torrentà, Carme Bertran-Noguer

**Affiliations:** ^1^Grup de recerca Salut i Atenció Sanitaria, University of Girona, Girona, Spain; ^2^Department of Neurology, Centre d’Esclerosi Múltiple de Catalunya (Cemcat), Hospital Universitari Vall d’Hebron, Barcelona, Spain; ^3^Grup d’Investigació Multidisciplinari d’Infermeria, Vall d’Hebron Institut de Recerca (VHIR), Hospital Universitari Vall d’Hebron, Barcelona, Spain; ^4^Expert Patient Program Catalonia, General Directorate of Health Planning and Research, Department of Health, Generalitat de Catalunya, Barcelona, Spain; ^5^Neurodegeneration and Neuroinflammation Research Group, Girona Biomedical Research Institute (IDIBGI), Salt, Spain; ^6^Girona Multiple Sclerosis and Neuroimmunology Unit. Neurology Department, Dr. Josep Trueta University Hospital and Santa Caterina Hospital, Girona-Salt, Spain; ^7^Department of Medical Sciences, University of Girona, Girona, Spain

**Keywords:** clinical research protocol, multiple sclerosis, health education, health literacy, patient empowerment, peer group, quality of life

## Abstract

**Introduction:**

Multiple sclerosis (MS) causes a progressive disability, which substantially impacts the quality of life (QoL). Health interventions that meet the needs and demands of people with MS are essential to minimize QoL impairment. Expert patient programs (EPPs) facilitate health-related empowerment through peer learning. Based on a previous focus group study, we designed an EPP for MS coordinated by nursing professionals for implementation in the different MS reference units of Catalonia (Southwestern Europe). This study aims to evaluate the effects on quality of life, disease-related knowledge, and self-management related to the health process of the participants of the Expert Patient Program Catalonia™ for people with multiple sclerosis (EPPC-MS).

**Methods:**

Pre-post intervention multicenter clinical study involving 12 groups of 12 participants: six groups including relapsing and six groups including progressive MS patients, with 144 participants from 7 MS reference units from all over Catalonia, organized in six teams. The intervention will consist of nine telematic learning peer-led sessions (one weekly session). The expert patient (EP) leading the sessions will be an individual with MS with disease-related knowledge, who will be further trained by nurses to lead the sessions. Study variables will be measured before and immediately after the intervention and 6 and 12 months after the end of the sessions and will include: QoL, emotional impact, activation of the person, MS-related knowledge, fatigue, habits and lifestyles, health services use, and program-related experience. Baseline characteristics considered will be sociodemographic data, date of MS diagnosis and type, family history, and treatment characteristics. Variables related to disease follow-up will be new relapses and characteristics and changes in the ongoing treatment. The number of sessions attended will also be collected. Study variables will be analyzed using a pre-post comparison.

**Discussion:**

Peer-led learning programs led by EP help empower people with chronic conditions and offer them tools to improve their autonomy and QoL. This study’s intervention will be performed remotely, offering advantages both for people with chronic conditions and the healthcare system regarding the facilitation of family and work conciliation, saving time, simplifying attendance to meetings, lowering costs, and using fewer material resources.

**Trial registration:**

NCT04988880 on September 22, 2021.

## Introduction

Multiple sclerosis (MS) is a chronic autoimmune degenerative disorder of the central nervous system with a variable clinical course characterized by a progressive disability, causing a substantial impact on quality of life (QoL) (1). Identifying and solving the needs and demands of individuals with MS is fundamental to minimizing their QoL impairment and improving their well-being (1). However, the needs and demands related to MS diagnosis and evolution generate a dynamic and complex scenario, requiring an individualized and high-quality healthcare planning focused on solving these needs (2). In this regard, patient-centered healthcare systems are more cost-effective and improve health literacy and patient participation (3). In addition, it is necessary to simultaneously incorporate a positive view of health that promotes individuals’ empowerment to solve health challenges (2).

Expert patient programs (EPPs) facilitate patient-centered healthcare delivery by improving individuals’ understanding of chronic diseases through knowledge transfer and exchange, thus promoting autonomy and self-care through a proactive approach, improving health indicators and reducing the use of healthcare resources (4). In addition, they facilitate the empowerment of people with MS and improve their well-being and overall perception of health (5, 6), enabling them to reorient their priorities, plan their activities, and choose their care and health preferences (7).

An Expert Patient Program of Catalonia™ (EPPC), consisting of a peer-led learning intervention, was included in the Health Plan of Catalonia 2011–2015 (Spain) and maintained in subsequent plans. The EPPC focused on self-responsibility, self-care, and promotion of people’s autonomy (8, 9). The Expert Patient (EP), a non-healthcare professional with the same chronic condition, leads the program sessions, developed in groups of 10–12 participants with the same long-term condition, guided by a nurse (4). EPs are people with significant knowledge of their disease and treatment in addition to self-management skills, who take responsibility for self-care and disease management, know how to identify and respond properly to symptoms, and acquire skills enabling them to manage the impact of the disease, resulting in improved QoL (10). The EPPC complements other health interventions led by healthcare professionals and is not currently implemented for the MS population.

In Catalonia, which has a population of over 7 million, more than 10,000 people have MS, of which almost 7,000 are women (11). A previous qualitative study based on focus groups including people with MS allowed to identify the topics to be addressed in the sessions, the ideal profile of the EP and the participants, the program’s design, and the potential benefits of a specific Multiple Sclerosis Expert Patient Program of Catalonia (EPPC-MS) (12). As a result of this pivotal study, the research team defined the final design and contents of the EPPC-MS, which will be implemented across the Catalan territory and remains to be evaluated for its ability to improve the empowerment and QoL of people with MS.

## Objectives

The primary objective of this study is to deploy the EPPC-MS across Catalonia. Secondary objectives are to evaluate the effectiveness of the EPPC-MS in reducing the emotional impact of MS and improving QoL, fatigue management, patient activation, habits and lifestyles, and MS knowledge of participants after completing the program. Additional secondary objectives are to evaluate the effectiveness of EPPC-MS in improving health outcomes according to the type of MS, the use of healthcare services by participants, the satisfaction of the EP and the participants with the EPPC-MS, and the long-term persistence of program benefits (at 6 and 12 months).

## Methods

### Overall study design

This research will be conducted using a pre-post intervention multicenter clinical study design to evaluate the territorial implementation of a specific EPPC-MS for people with MS. The study is planned for development during a period of 36 months (January 2021–December 2023). The common goal of this kind of study is to find whether a health intervention affects the participants and their clinical variables and health-related outcomes (13). The intervention sessions will be conducted remotely using Microsoft Teams platform to avoid face-to-face meetings during the current pandemic situation (SARS-CoV-2) as part of a risk mitigation strategy (14). EPPC managers will provide this platform linked to the Health Department of Catalonia, who will endorse its use. Each intervention will consist of nine weekly sessions, lasting an average of 3 months.

This study protocol adheres to the Standard Protocol Items: recommendations for Interventional Trials (SPIRIT) guidelines^1^ for reporting study protocols.

### Study setting

**Figure 1:**
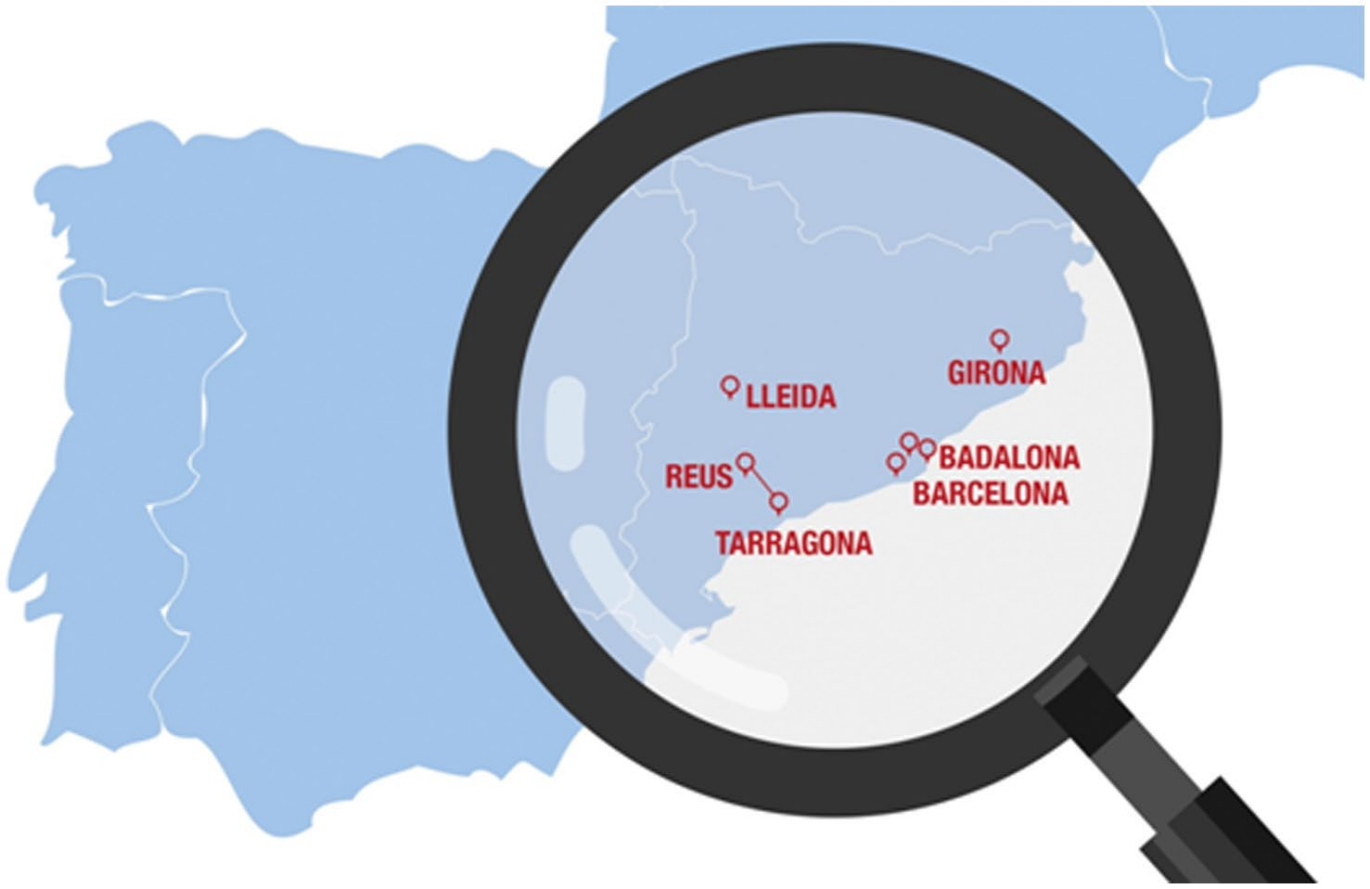
Location of the seven participating multiple sclerosis units. The line between the hospital in Tarragona and the hospital in Reus indicates that these 2 units will participate as a joint team, resulting in total of six participating teams.

The Catalan healthcare system provides free and universal healthcare to its over 7 million population. The estimated 10,000 individuals who live with MS in Catalonia receive care in multidisciplinary reference MS units. The study will be carried by six teams from 7 MS units located across Catalonia: one from Girona, three from Barcelona, one from Lleida, and two from the Tarragona area (Tarragona unit and Reus unit) pariticipating as a joint team (Figure 1). The participating MS units are the main multidisciplinary reference units of the territory for individuals diagnosed or suspected of demyelinating disease and specifically MS. The study will start after the approval of each local Clinical Research Ethics Committee.

### Participant and unit timeline

**Figure 2:**
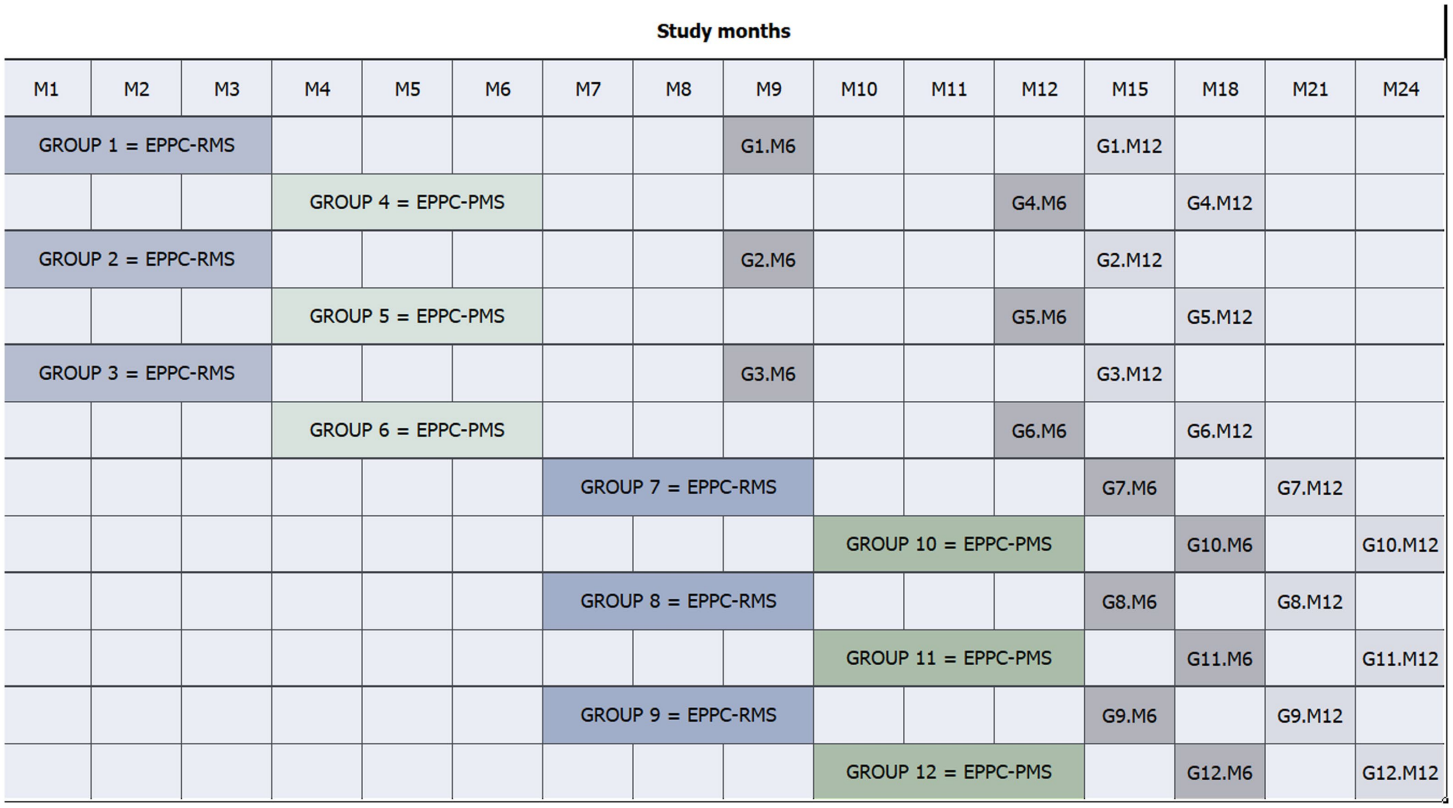
Flow diagram and timeline of participants. BICAMS, Brief International Cognitive Assessment for Multiple Sclerosis; HADS, Hospital Anxiety and Depression Scale; M, month; MS, multiple sclerosis. *Participant candidates with BICAMS and HADS data obtained in the routine neurorehabilitation screening will not be required to sign an informed consent for the screening procedure, only to participate in the study.

Each unit will identify and recruit two groups of people with two forms of MS: one group of people with relapsing MS and one with progressive MS, resulting in a total of 12 groups. The program will be implemented in four consecutive phases, including three groups of participants from 3 MS units per phase that will undergo the 9-week intervention simultaneously. After completing the intervention in Phase 1, the same 3 units will start Phase 2 with the remaining three groups in any order (e.g., first the relapsing group and then the progressive one). Hence, 3 MS units will participate in the first two phases. Immediately after the first two phases are completed, the three remaining units will start the intervention in their groups in Phase 3 and, after completing the three groups, in Phase 4. Each group will be invited to attend a follow-up session 6 and 12 months after the end of the intervention to share any changes on their daily habits resulting from the learning program, and the application of disease management strategies (see Figure 2).

### Selection of participants and EPs

Patients with a diagnosis of MS treated in the participating MS units will be eligible to participate as participants or EPs in the deployment of the EPPC-MS. All members of the MS multidisciplinary teams will identify participant and EP candidates according to their criteria based on their MS-related knowledge and ability for self-care and self-management, and will invite them to participate. The teams coordinate the process of selection and assessment of participants’ inclusion criteria, including a visit with a trained neuropsychologist to administer the Brief International Cognitive Assessment for Multiple Sclerosis (BICAMS) (15) and Hospital Anxiety and Depression Scales (HADS) (16). Furthermore, the teams initiate and coordinate all the steps for the selection of EPs among pre-selected candidates. A nurse [i.e., the study’s principal investigator (PI)] will coordinate and provide support for these processes to the participating MS units, and will monitor the process to ensure protocol compliance.

Eligible participants must meet the following inclusion criteria: (1) be ≥18 years old; (2) have reported the need for support for disease self-management or to improve MS-related knowledge; (3) confirm availability to attend at least 80% of the sessions; (4) sign an informed consent. Patients meeting any of the following exclusion criteria will not be eligible: (1) be unable to speak or write in Spanish or Catalan; (2) have aphasia or an auditory disorder preventing them from interacting with the group; (3) have a severe cognitive impairment, defined as a score of 1.5-fold the standard deviation below the population mean in one of the three subtests of the Spanish version of the BICAMS (15); (4) have severe emotional impairment, defined as a score above 11/21 on any of the two Hospital Anxiety and Depression Scale (HADS) subscales (anxiety and depression) (16); and (5) have any psychopathological comorbidity or mental disorder diagnosis that may restrict their interaction with the group.

To be eligible as EPs, individuals must meet the same inclusion criteria as participants. However, they must lack the need for support for disease self-management and should have MS-related knowledge. Healthcare or educational professionals and individuals meeting participants’ exclusion criteria will be ineligible as EPs. Furthermore, EPs are selected among patients diagnosed before 2018 to ensure sufficient disease-related knowledge based on their own experience and interactions with the health care system and professionals. Additionally, EPs are selected among patients showing the ability for self-management and self-care and those with a positive perception of the disease. No objective instrument or scale will be used to assess EP inclusion criteria. However, health care professionals from the MS units follow patients closely, getting to know them and, therefore, can appraise the patients’ characteristics and skills. Furthermore, the selection of patients diagnosed before 2018 ensures a long-term relationship with the multidisciplinary teams. Irrespective of these criteria, potential EP candidates could meet other characteristics, such as information and communication technologies skills, communication and leadership skills, and comprehensive experience and knowledge of the health process. The information and communication technologies skills are partly appraised during contacts with the MS units using electronic communication tools, such as email or other platforms, and by evaluating the use of reference information websites. During the selection process, EPs are informed that their participation will be altruistic and should expect no compensation.

Each of the participating teams will select three EP candidates, who will be scheduled for interviews with the PI and the institutional managers of the EPPC to further assess their adequacy to fulfill the EP role. Disease-related knowledge will be assessed using questions from the disease knowledge, habits and lifestyle, and quality of life questionnaires used to evaluate the program, and candidates will be engaged to speak about self-management, self-care, and their future role as EP to assess their predisposition, leadership skills, and communication abilities. This strategy for EP selection has been used previously in other versions of the program (17). Moreover, the interviews will be conducted remotely using the same platform that will be used for the program sessions to confirm the ability of the EP candidates to use it. The PI and EPPC managers will select one EP per team, who will be provided with the session materials and will be trained for their role. The training sessions will allow us to confirm the adequacy of the EPs and, if necessary, the selection may be reconsidered.

### Sample size

Sample size is estimated based on the deployment of the EPPC-MS across Catalonia (primary outcome). Sample size was calculated by prioritizing those units willing to participate in the study and interested in implementing the program in the future to offer it to the people with MS followed up at the unit regularly. Of the 7 units willing to participate, the 2 units located in the Tarragona area (Tarragona and Reus units) will particpate as joint team, resulting in a total of six participating teams. Each MS team will include two groups of people with MS, of which one group will include people with relapsing MS and the other group people with progressive MS, resulting in a total of 12 groups, with six groups for each MS type. Based on the methodology used in the program, the recruitment target will be 12 people with MS per group (144 individuals in total). We expect this group size will compensate for possible losses during the intervention and ensure a minimum of 8–10 participants per group throughout the program (96–120 participants) (4). We estimated this sample size based on the number of people with MS in Catalonia (⁓10,000) and the number cared for at each of the MS units. Ideally, the exact calculation of the sample size required to assess the study’s secondary outcomes (EPPC-MS effects) should be based on the results of a pilot study testing the program’s effects in a smaller sample of patients. However, this study has not yet been completed. If the 12 groups are insufficient to reach an appropriate sample, the participating investigators will contact the Ethics Committees to request their approval to expand the number of groups.

### Recruitment

Participants: follow-up of people with MS involves therapeutic support by the multidisciplinary team during clinical visits at the unit facilities (i.e., regular follow-up visits), the day care clinic, or through non-scheduled consultations for any reason (i.e., needing additional care due to treatment secondary effects or disease symptoms, information, and support). After completing a routine follow-up visit by a healthcare professional, people with MS self-reporting the need for support for disease management or the need to improve MS-related knowledge will be invited to participate in the study. To ensure that the target sample size is reached, healthcare professionals, members of the MS multidisciplinary teams may actively identify candidates by asking people with MS about their need for additional support and information for disease management. People with MS initially willing to participate will be referred to the study coordinator at each participating center to receive complete information about the study.

**Figure 3:**
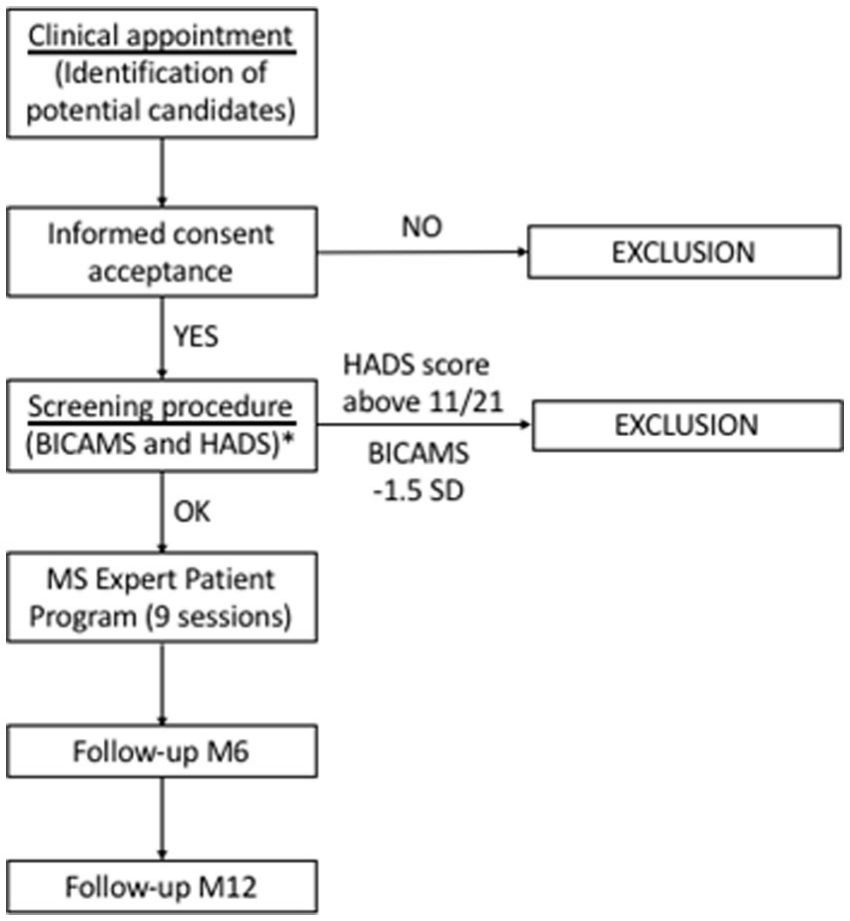
Expert Patient Program timeline. EP, expert patient; EPPC-MS-Relapsing and EPPC-RMS, expert patient group including participants with relapsing multiple sclerosis; EPPC-MS-Progressive and EPPC-PMS, expert patient group including participants with progressive multiple sclerosis; G1 to G12, expert patient groups 1 to 12; M, month.

Neuropsychologists within the MS multidisciplinary teams will assess the inclusion and exclusion criteria of participant candidates. To assess severe cognitive and emotional impairment (exclusion criteria), a trained and experienced staff in assessing cognitive and emotional impairment will screen participant candidates using the BICAMS (15) and HADS (16) instruments. These tests are part of the routine neurorehabilitation screening procedure, but may be administered to other participant candidates outside the routine screening that require a specific consent. Before the study starts, a senior neuropsychologist will lead an update session for all neuropsychologists of the participant centers to ensure uniformity of data collection (18). Eligible candidates will receive the patient information sheet and be asked to sign a written informed consent before assessing the selection criteria, such as cognitive and emotional impairment (when required). The participant timeline summarizes the recruitment process (Figure 3).

Expert Patient candidates will be selected among the MS population followed up at each unit and according to the eligibility criteria. The study PI will evaluate the knowledge of EP candidates to confirm their eligibility. Additionally, people with MS who participated as EP in the pilot study testing the program effects or other MS units may be invited to participate again as EPs in this study.

### Intervention

The intervention aims to reinforce positive knowledge concisely using plain language that all participants can understand. Throughout nine 90-min sessions (one per week) led by an EP, participants in the group will share their knowledge and experiences on all aspects of MS and its self-management. The organization of session and its contents are summarized in Table 1.

**Table 1:** Expert Patient Program Catalonia-Multiple Sclerosis (EPPC-MS): sessions and contents.

Session	Content description
1. Introduction	Presentation of group members, program objectives, session contents, session development, and questionnaires
2. The disease	What is multiple sclerosis? Concept, causes, types of MS, and diagnosis
3. Signs and symptoms	Relapses (relapsing MS), progression (progressive MS), and fatigue
4. Treatment	Existing treatment, future treatments, treatments during pregnancy, and alternative therapies
5. Health promotion	Healthy lifestyles: nutritional facts, activity and exercise, tobacco, vitamin D and sun exposure, and obesity
6. Taboo subjects	Urinary and fecal incontinence, sexuality, and sexual dysfunction
7. Emotional impact and relationships	Coping with the disease, work and family relationships, and available resources for emotional support
8. Social environment and quality of life	Rehabilitation and physical aspects, social resources, and benefits
9. Program finalization	Wrap-up session

The study’s PI is a nurse specialized in MS and will train the EPs and support them throughout the intervention, together with professionals in each of the participating units. Before starting the intervention, the study’s PI will train the EPs in a presentation of all the learning materials and contents of each session. Furthermore, using role-playing activities, the nurse PI will ask example questions related to the contents of each session to the EPs to be answered jointly. The training and activities aim to expand the EPs’ knowledge of the sessions’ contents and gain confidence to lead and answer the questions.

Throughout the intervention, a professional nurse in the MS team and the EP will meet before each session to prepare the contents and solve any doubts from the previous sessions. In all sessions, two nurses, on from the participating team and one member of the EPPC panel of directors, will act as an observers, provide help and support to the EP if needed, and assess each session jointly with the EP at the end.

Sessions are divided into two blocks: a first block with a theoretical introduction to focus on the day’s topic, conducted by the EP (30 min), and a practical block (60 min). A previous pivotal study characterized the topics addressed in the sessions (12). After the first block introducing the topic, the EP will encourage interaction, facilitating the communication of doubts, questions, or experiences related to the contents, which will be solved or answered by participants or collectively through discussion within the group (practical block). This intervention strategy with an EP leading the session and the participation of all the group members aims to empower them to gain control over their health situation. The knowledge acquired will come from the information provided by the EP and the knowledge resulting from the life experiences regarding MS of all the participants involved in the intervention.

Participants will complete questionnaires according to the study schedule (prior and immediately after the intervention, and at the evaluation periods 6 and 12 months after the end of the intervention) to assess the secondary outcomes. The completed questionnaires will be collected at post-intervention sessions (at 6 and 12 months), allowing participants to clarify concepts and expose doubts regarding the contents of the previous nine sessions.

To ensure adherence, the platform will send reminders before each session and, if necessary, sessions will be rescheduled.

### Outcomes

The primary outcome of this study is the deployment and implementation of the EPPC-MS in the participating MS units across Catalonia. The feasibility of the EPPC-MS implementation will be appraised in terms of the correct inclusion of the six participating teams and the successful execution of all the procedures. Participation in the study entails sufficient training of professionals at MS units for the correct identification and recruitment of participants and EPs and for appropriate data collection and monitorization of study procedures. At the end of the study, the participating units will be able to implement the EPPC-MS independently and autonomously based on the methodologies of the EPPC-MS.

Secondary outcomes are those related to the effectiveness of the program and include changes in QoL, measured using the Multiple Sclerosis Quality of Life 54 items (MSQOL-54) questionnaire (19, 20); emotional impact, measured using the HADS (16); fatigue management, measured using the Fatigue Severity Scale (FSS) (21); patient activation and engagement, measured using the 13-item Patient Activation Measure (PAM-13) (22); patient knowledge, measured using the EPPC specific questionnaire about MS-related knowledge; individuals’ habits and lifestyles, measured using the EPPC specific questionnaire about habits and lifestyles; use of healthcare services, such as visits to the MS unit, visits to the general physician (GP), and visits to the accident and emergency (A&E); and Patient Reported Satisfaction (i.e., experience), measured using the EPPC-specific questionnaire about patient-reported experience measures (PREMs), designed *ad hoc* for this study. Except for the latter, we will compare the scores obtained before the intervention and at post-intervention timepoints (immediately after the intervention and at 6 and/or 12 months after the end of the intervention). The participants and the EPs will answer the patient-reported satisfaction questionnaire, and scores will be compared immediately after the intervention and at 6 and 12 months.

### Study variables and data collection

**Figure 4:**
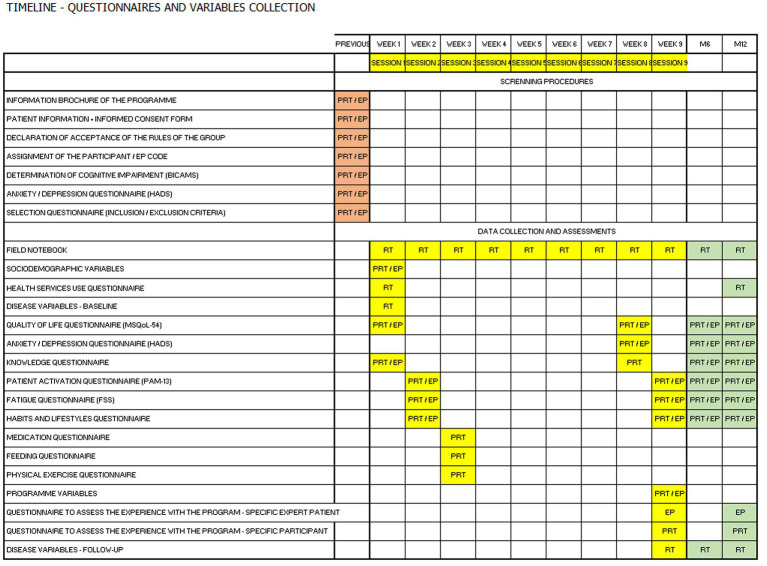
Assessments and data collection timeline. BICAMS, Brief International Cognitive Assessment for Multiple Sclerosis; EP, expert patient; FSS, Fatigue Severity Scale; HADS, Hospital Anxiety and Depression Scale; MSQoL-54; Multiple Sclerosis Quality of Life-54 items; PAM-13, patient activation measure; PRT, participants; RT, research team.

Data will be collected according to the study timeline depicted in Figure 4. Baseline sociodemographic characteristics of EPs and participants, including age, gender, marital status, family support, educational level, and employment situation, will be collected during the recruitment visit. Disease variables, including, among others, date of diagnosis, type of MS, MS treatment, and disability, will be collected from the clinical records before the intervention (Week 1) and after the intervention (Week 9 and at 6 and 12 months after the end of the intervention). Variables related to the use of healthcare services include the number of visits to the MS unit (both scheduled routine follow-up visits and unscheduled visits), primary care visits, and A&E department visits during 1 year before and after the intervention, and will be collected prior to the intervention (Week 1) and after the intervention (Month 12), respectively, by the research team using a questionnaire. This information is routinely registered during visits to clinics and healthcare consultations. It is made available by the health registry of the region to the healthcare institutions after institutional authorization from the Ethics Committee of each center. Before the study starts, all the healthcare professionals involved will receive an update session led by the study’s PI regarding data entry to ensure uniform data collection (18).

Patient-reported outcomes (PROs) and PREMs evaluating the impact of the intervention (secondary outcomes) will be assessed using specific instruments (i.e., questionnaires) according to the study timeline (Figure 4). Except for participants and EP experience with the program, which will be evaluated after the intervention, questionnaires will be self-administered prior to the intervention (Weeks 1–3) and after the intervention (Week 9, Month 6, and Month12). Pre-intervention questionnaires will be distributed within the first 3 weeks considering the contents of each session. Hence, questionnaires assessing fatigue and lifestyle will be administered in session 2 (Week 2) and the corresponding contents will be presented in sessions 3 and 5, respectively. The Research Electronic Data Capture (REDCap) platform will automatically send the questionnaires and instructions for completion, and participants will complete them online on the platform’s site. The platform automatically resends the questionnaires weekly during three consecutive weeks in case answers are not received. Moreover, it allows real-time monitoring of the questionnaires received and the PI may resend the questionnaires manually and contact participants to solve any issues regarding questionnaire completion on the platform’s site.

The study’s PI, a nurse, will maintain a field notebook to record the basic information for each session, including participants, the session’s contents, the group, and the MS reference unit. The PI will additionally collect participants’ comments and questions related to the session’s topic and final comments regarding each session development. The EP will answer all questions collected as unanswered during each session in the next. Furthermore, the EP will clarify any inappropriate or inaccurate information shared during sessions in the next session, with the support of the research team.

#### Quality of life

The Spanish validated translation (20) of the MSQoL-54 (19) is a self-administered questionnaire with a completion time of approximately 15 min and a Cronbach alpha ranging 0.75–0.96 for the different scales. It consists of 54 items, of which 36 correspond to the generic Short Form-36 Health Survey questionnaire and 18 are specific to MS. 52 items are classified in 12 dimensions. The remaining two individual items measure changes in health status (comparing current health and health during the previous year) and satisfaction with sexual function. In addition to the scores of the dimensions and individual items, two composite scores corresponding to mental and physical health subscales are obtained. The MSQoL-54 questionnaire is widely used and is, therefore, a relevant tool for assessing the impact of the EPPC-MS on participants’ QoL.

#### Emotional impact

The HADS (16) and the Spanish version elaborated by Herrero in 2003 (23) specifically assess the level of anxiety or depression of an individual in the last 7 days. Several studies have validated the translated version in the Spanish population, yielding Cronbach alphas of 0.87–0.90 (24). It consists of a 14-item self-administered questionnaire, including an anxiety and a depression subscales, with seven items each. For each subscale, the scores range from 0 to 3 (0 being the minimum affectation and 3 the maximum) for each item, whereby a 0–7 score indicates no disorder, 8–10 is a questionable case, and a score ≥ 11 indicates the presence of anxiety or depressive disorder. HADS is a useful tool for detecting individuals with a neuropsychological condition requiring referral to a specialist for assessment and care (exclusion criteria) and evaluating the program’s effectiveness on MS-related emotional impact.

#### Fatigue

The FSS (21) and its Spanish translation by Tola in 1998 (25) is a 9-item self-administered questionnaire, rated on a 1–7 scale (1 being “strongly agree” and 7, “strongly disagree”) assessing the impact of fatigue on a person’s daily life. The Cronbach alpha was 0.81 for its English version. This tool will enable the evaluation of the program’s impact on fatigue self-management.

#### Patient activation (engagement)

The PAM-13 and its validated Spanish version by Moreno-Chico in 2017 (22) is an instrument assessing knowledge, skills and people’s confidence in self-management of their health and medical care. The validation of the Spanish version showed an Item Separation Index (i.e., comparable to Cronbach alpha, whereby values ≥0.7 show a differentiated scale between individuals and items along the latent trait) of 6.64 (22). PAM-13 consists of a self-administered questionnaire of 13 items, rated on a 1–4 scale (1 being “strongly agree” and 4 being “strongly disagree”). PAM-13 is a useful tool to assess patients’ confidence regarding disease self-management and will enable evaluating the program’s impact on the activation and involvement of participants.

#### Knowledge of MS

Given the lack of validated questionnaires in Spanish assessing MS-related knowledge, the expert panel of the EPPC developed a specific questionnaire to assess MS-related knowledge. This questionnaire assesses disease-related knowledge and specific aspects essential to empower MS patients identified as the target population through a qualitative study of focus groups (12). This questionnaire, which has not been validated yet, was designed based on the previous pilot qualitative study (12) and will assess the impact of the program on the acquired knowledge.

#### Habits and lifestyles

The EPPC coordinating panel developed an MS-specific questionnaire to assess habits and lifestyles. This questionnaire awaits validation, but has already been used in other program variants, demonstrating its usefulness in assessing changes due to participation in this program (4, 26).

#### Participant-reported experience and expert patient-reported experience

The EPPC coordinating panel developed a specific questionnaire to assess satisfaction related to their participation and experience with the intervention. This questionnaire has already been used in other variants of the program, demonstrating its usefulness in assessing participants’ experience in this program (4, 17, 26).

### Data management

Study data will be collected and managed using REDCap electronic data capture tools hosted at Vall d’Hebron Research Institute (VHIR). The REDCap (Research Electronic Data Capture) is a secure, web-based software platform designed to support data capture fro research studies. The REDCap provides (1) an intuitive interface for validated data capture; (2) audit trail for tracking data manipulation and export procedures; (3) automated export procedures for seamless data downloads to common statistical packages; and (4) procedures for data integration and interoperability with external sources (27, 28). Each participant will be assigned a code for de-identification. No data will be shared. Each site will send the results to the coordinating center, and the PI will be responsible for guarding the database, which will be saved until the analysis is complete.

### Contact details of participants

Each unit will provide minimum and essential contact details to allow communication between the program coordinating team and EP or participant candidates. Data will be stored by the coordinating team in a different database than the study’s database, and will be destroyed after the intervention is completed. Only the PIs at each participating unit will have access to the database. These data must include the code assigned to each individual, name and surname, telephone number, and e-mail address to send them the program questionnaires and instructions to connect to the Patient Expert Catalonia Program platform.

### Variables and questionnaires

The REDCap is an electronic data capture software and workflow methodology we will use to design the research database (27, 28). REDCap, developed by Vanderbilt University, provides institutional licenses to various clinical research institutes worldwide. In this study, the Basic Statistics Unit (UBE) associated with the Vall d’Hebron Research Institute (VHIR) of the Vall d’Hebron Barcelona Hospital Campus will manage the institutional license provided and guarantee security of the data and the computer support for databases created with this system. Specifically, in this study, the UBE will support the study’s PI to create the database and, through a collaboration agreement, will perform the statistical analysis of this study’s outcomes. The coordinating team will enter all data related to the questionnaires into the study database using REDCap as participants complete the questionnaires. The centers’ research teams will provide all clinical or disease-related variables with a code for each participant, without any sensitive data allowing the identification of study participants. Therefore, their identity will remain blind to the research team of each center, who will have access to the REDCap platform for data entry. As mentioned above, each center will send the results to the coordinating center through the platform. The nurse principal investigator will keep the database and save it until the end of the analysis related to the research project.

### Statistical analysis

Statistical analyses will be performed on the intention-to-treat (ITT) population, defined as the population of patients starting the intervention, irrespective of program completion. Continuous variables will be described as mean and standard deviation (SD) or, in case of non-compliance with normality criteria, median and interquartile range (IQR), and categorical variables as absolute frequency and percentages. The non-parametric U Mann–Whitney test for independent samples and the Wilcoxon test for dependent samples will be used to analyze the relationship between a categorical variable and a continuous variable. The Chi-squared test or the Fisher’s exact test will be used to analyze the relationship between two categorical variables. For the secondary outcomes, changes in the study variables throughout time (i.e., before and after the intervention) will be analyzed using an ANCOVA or linear mixed model to etimate changes in effect size. No methods to handle missing data (i.e., imputation) will be applied. A significance level of 5% will be considered. Data will be analyzed using the latest version of the Statistical Package for the Social Sciences (SPSS) software (IBM, Armonk, New York, United States).

### Monitoring

A study monitor will visit the participating centers before starting recruitment to ensure that they have the human and material resources and the necessary training for their satisfactory participation in the study and in accordance with the protocol. The research staff of each unit will attend a training session before the recruitment starts and a monthly meeting to verify uniformity in data collection. The role as observer of the study’s nurse PI during the program will facilitate the prompt detection of protocol deviations and data collected in the field notebook will allow the research team to implement the necessary correcting actions. For example, unanswered questions during each group session will be collected in a field notebook by the research team and answered in the next session. Furthermore, inappropriate or inaccurate information shared during sessions will be clarified by the EP in the next session, with the support of the research team.

A data monitoring committee was deemed unnecessary in this study, as the intervention was anticipated to cause no harm to any of the participants. The EPPC has been implemented for other chronic conditions, such as heart failure, chronic obstructive pulmonary disease, Chagas disease, and intestinal inflammatory bowel disease, demonstrating the safety of peer-led educational interventions (17, 26, 29). In this regard, this study will not include a safety assessment, as the intervention is not expected to generate any related adverse events. Nevertheless, changes in the HADS (23) subscales scores may indirectly provide valuable safety information and will be monitored. Furthermore, a negative impact of the intervention may lead to missing sessions, and we will contact participants with missing sessions to provide support. If the investigators detect any issues, the participant will be referred to the relevant specialist.

### Ethics statement

The study protocol (version 2.0) was approved by the Clinical Research Ethics Committee (CEIM, *Comité de Ética de la Investigación con medicamentos*) of the Hospital Universitario de la Vall d’Hebron with protocol number PR(AG)334/2021 on June 26, 2021. It was registered at clinicaltrials.gov (NCT04988880) on September 22, 2021. This study will be conducted following the foundations of the Declaration of Helsinki and the current legislation regarding confidentiality of personal data in clinical studies: the Spanish Personal data protection regulations, the Organic Law 3/2018, of 5 December, on the Protection of Personal Data and the guarantee of digital rights (BOE 6 December 2018, applicable from 7 December 2018).

The CEIm that approved the protocol performs a scheduled follow-up of the studies by requesting information regarding recruitment, study development, and modifications to the PI. Furthermore, this study’s PI is committed to update the study information (i.e., protocol and status) on the registry (Clinicaltrials.gov).

Participants will be informed of the voluntary nature of the study, and their participation will not be compensated. Participants will have time to decide whether they will take part in the study. Informed consent forms will assure anonymity and confidentiality regarding adaption, presentation, and data publication. Participants will also be informed that they can withdraw from the study without having to give a reason. All participants will sign informed consent forms to confirm their acceptance to participate in the study. They will also be informed verbally by the study coordinator and researchers before any procedure. The authors confirm that no patient/personal identifiers will appear in any publication, ensuring the anonymity of the study participants.

### Data access

Study investigators will have access to their local study data during the research. After data collection is completed, only the manuscript authors and the study’s PI will have access to the data, stored in a trusted digital repository. After study completion, the anonymized data will be made available for future research. Patients provided informed consent regarding the use of data obtained in this study in future research.

### Dissemination planning

The results of the research study will be published, within the framework of the requirements of the doctoral program, in journals indexed in the JCR. The research team will disseminate the results of the study at national and international scientific conferences in the fields of neurology, MS, and health promotion. Importantly, the EPPC-MS implemented in Catalonia will become available to the target population (MS patients) through disseminating the nurse-led intervention to other MS units in Catalonia. Moreover, any opportunities to improve its effectiveness identified in this study may be implemented before the dissemination of the intervention. The study protocol, statistical codes, and datasets will be available upon reasonable request.

## Discussion

This multicenter clinical study aims to implement an intervention in patients with MS and evaluate its effects on QoL, disease-related knowledge, and self-management related to the health process of the participants. The intervention consists of educational discussion sessions led by an EP and coordinated by a nurse within the Expert Patient Program of Catalonia framework.

Interventions involving EPPs have proven to be useful before, as well as other patient support programs with similar goals (4–6). However, they have all been based on in-person strategies, where all participants meet and interact simultaneously and in the same place. The leadership of the nursing coordinating team has facilitated the adaptation of the study to the current COVID-19 pandemic situation, with sessions that will be carried out using a telematic format. This format is new compared with previous studies. At the same time, it minimizes potential risks associated with personal contact and there is less use of material resources to carry out the program (rooms, spaces, etc.). Despite the decreased COVID-19 restrictions and risks since this intervention was envisioned, the telematic format is still an advantage for health centers owing to the low economic cost for participant sites, and offers many advantages for program participants as well. First, it avoids unnecessary travel, facilitating family conciliation. On the other hand, meeting attendance is made easier for those people with reduced mobility, some type of difficulty of access, or those who live farther away from the site. For working people, the potential impact of their attendance on their work life is minimized. Because of all this, the program will have a direct influence on individuals and their lifestyles, on their education and knowledge of health, and on the possible inequities in access to the health system, which, in turn, will have a direct impact on social health determinants affecting the individual.

However, it must be considered that there may be a digital gap in certain groups of patients; therefore, not everybody will potentially benefit from a remote strategy, and some patient groups may be unintentionally excluded due to the intervention telematic format. Upon completion of this study, the EPPC-MS will become available to all MS patients in Catalonia. Future editions of the EPPC-MS may consider other intervention formats, including face-to-face and hybrid formats, to reach all the target population. In this regard, the EPPC for other chronic diseases, such as heart failure and chronic obstructive pulmonary disease, was implemented in a face-to-face format and therefore, we anticipate that adapting the EPPC-MS to this format will be feasible (30, 31).

The EP figure favors health literacy. This term encompasses all health-related knowledge, skills, and experiences that make a non-health professional individual an expert in their state of health and how to take care of themselves (32). Health literacy is effective in promoting health in patients with chronic diseases, such as MS. It provides knowledge on the disease, symptoms, and care that each patient needs and favors treatment adherence. Consequently, health literacy entails empowerment over your health, making patients go from a passive role to a much more active one, who will thus take care of getting the best medical care possible, managing their disease and, ultimately, will be much more committed to their health condition (10). Health-related empowerment directly impacts QoL, symptom management, emotional status, and the person activation. Regarding the healthcare system, higher empowerment can also reduce the use of health services (33).

Health literacy is beneficial for healthcare professionals as well. Knowing the degree of health literacy in their patients may help the nursing team to identify those at a higher risk of suffering misunderstandings or lack of treatment adherence (34).

This study is aligned with the current Health Plan for Catalonia 2021–2025 (35) and with the WHO global strategy on integrated people-centered health services (36). These especially emphasize the need to promote autonomy and adaptation of healthcare systems to provide the best possible care to the elderly or people with chronic conditions.

This study assessing the intervention effectiveness and EPs’ and participants’ satisfaction, will enable to identify opportunities to improve the EPPC-MS in its future nurse-led implementation throughout the Catalan territory. Nevertheless, the program may benefit from further research aiming to develop validated instruments to evaluate the suitability of candidate EPs regarding knowledge and leadership skills, and the disease-related knowledge acquired by participants compared to non-participants with MS.

### Limitations

Health regulations and recommendations owing to the COVID-19 pandemic may alter the study’s schedule due to the restrictions at the MS units on participants’ attendance to assess inclusion and exclusion criteria. Being a study lasting more than 12 months, the elimination of the knowledge-induced response of studied participants is guaranteed (37). With respect to session attendance and missing data, the program will ensure attendance by sending reminders to confirm it and, if necessary, re-scheduling sessions. Furthermore, the questionnaires’ receipt from each participant will be monitored and reminders will be sent to ensure collection of data at all timepoints. Regarding evaluation instruments, a Spanish version of the BICAMS (15) battery has been developed for the Argentine population (15). To date, we do not have scales for the population of Spain. Nevertheless, the demographic characteristics of the sample used in Vanotti’s study are likely extrapolable to the population’s demographic characteristics in the present study. Likewise, instruments evaluating MS-related knowledge and habits and lifestyle are still missing. Therefore, this study will evaluate these variables using questionnaires developed by the EPPC and awaiting validation. In this regard, an objective instrument to evaluate EPs’ skills and knowledge is missing and therefore, existing differences among EPs may influence this study’s results. However, we believe that EP planned training before starting the learning sessions may partly even any potential differences.

Regarding the study’s design, an approach to the study phenomenon through a pragmatic clinical trial was considered in the planning phase. However, a larger sample size would be needed to establish comparability, which we deemed difficult to achieve. Of the 7,000 people with MS in Catalonia, the population with severe cognitive or psycho-emotional impairment must be ruled out, considerably decreasing the eligible population. Furthermore, this study’s main purpose is to implement the innovative intervention, and therefore, a pre-post intervention study design was chosen. In addition, the main goal is to evaluate an innovative practice rather than to establish causal relationships. The intervention aims to improve QoL and self-management skills regarding the health process and the implementation of a feasible health intervention within the services catalog of the respective units. Regarding the telematic nature of the intervention, the research team will provide informatic support, but will not provide electronic devices, hampering the participation of eligible people with MS who do not own a suitable electronic device. This limitation, together with limited digital skills, may be particularly relevant for elderly candidate participants. Moreover, it may bias patient selection and consequently, the results from this study may not reflect the outcomes obtained in a face-to-face implementation modality. The study will train the units to provide standardized and programmed peer support groups to people with MS.

## Conclusion

This protocol describes a pre-post intervention multicenter clinical study in patients with MS, which will be carried out in different MS reference units of Catalonia through an expert patient program with remote working sessions. The study will allow the evaluation of the impact of the program on participants’ QoL, disease-related knowledge, and self-management of MS.

## Ethics statement

The studies involving human participants were reviewed and approved by Clinical Research Ethics Committee of the Hospital Universitario de la Vall d’Hebron, PR(AG)334/2021. The patients/participants provided their written informed consent to participate in this study.

## Author contributions

MR-S: conceptualization, data curation, funding acquisition, investigation, methodology, project administration, resources, software, visualization, and roles/writing – original draft. PA-B: conceptualization, funding acquisition, investigation, methodology, resources, software, supervision, validation, visualization, writing – review, and editing. CB-F: conceptualization, data curation, investigation, methodology, software, supervision, validation, visualization, roles/writing – original draft, writing – review, and editing. CC-M, MA, EA, RM-D, XM, JS-G, LR-T, and CB-N: conceptualization, data curation, investigation, methodology, supervision, validation, visualization, roles/writing – original draft, writing – review, and editing. All authors contributed to the article and approved the submitted version.

## Funding

This study was promoted by the MS unit at Vall d’Hebron Hospital Campus and had no external sponsor. The PhD candidate/first author was supported through the Strategic Plan for Research and Innovation in Health 2016–2020 (PERIS) (ref. BDNS 542793) funded by the Health Department of Catalonia. This study had been partially funded by the Official College of Nurses of Barcelona (www.coib.cat) as part of the Nurse Research Projects Grants (PRN-475/2021). None of the funders were involved in the design of the study, manuscript writing or data collection, and will not be involved in data analysis or interpretation and manuscript writing in the future. The only funders’ requirement is that any publications associate with this study must be open access and deposited in an institutional repository.

## Conflict of interest

MR-S received speaking or consulting honoraria, participated in scientific activities organized by Merck, Teva, Biogen, Novartis, Sanofi-Genzyme, Celgene, EXCEMED, and Roche, and was awarded the ECTRIMS MS Nurse Training Fellowship Programme and the Strategic Plan for Research and Innovation in Health 2016–2020 (PERIS). CC-M has received support for attending congresses from sanofi, merk, teva and novartis and is funded by a fellowship from the Departament de Salut de la Generalitat de Catalunya [SLT017/20/000115, 2021]. MA received speaking or consulting honoraria, participated in scientific activities in the last 2 years organized by Bristol-Myers Squibb/Celgene. XM received speaking honoraria and travel expenses for participation in scientific meetings, has been a steering committee member of clinical trials or participated in advisory boards of clinical trials in the past years with Abbvie, Actelion, Alexion, Biogen, Bristol-Myers Squibb/Celgene, EMD Serono, Genzyme, Hoffmann-La Roche, Immunic, Janssen Pharmaceuticals, Medday, Merck, Mylan, Nervgen, Novartis, Sandoz, Sanofi-Genzyme, Teva Pharmaceutical, TG Therapeutics, Excemed, MSIF and NMSS. JS-G received speaking or consulting honoraria and attended scientific activities in the last 2 years organized by Merck, Teva, Bial, EXCEMED, Biogen, Celgene, Novartis, Sanofi-Genzyme, and Roche; and is the director of the “Revista de Neurologia” (Neurology Journal) and an editorial board member of the Multiple Sclerosis Journal. LR-T has received speaking or consulting honoraria, attended scientific activities organized by Merck, Teva, Biogen, Novartis, Sanofi, Roche, Bristol-Myers-Squibb, Almirall, and Mylan, and participated in advisory boards organized by Sanofi, Merck, Roche, Biogen, Novartis, Bristol-Myers-Squibb, and Almirall. PA-B, the coordinator of the Expert Patient Program Catalunya. CC-M has received support for attending congresses from Bristol Myers Squibb, Sanofi, Merck, and Novartis and is funded by a fellowship from the Departament de Salut de la Generalitat de Catalunya [SLT017/20/000115, 2021].

The remaining authors declare that the research was conducted in the absence of any commercial or financial relationships that could be construed as a potential conflict of interest.

## Publisher’s note

All claims expressed in this article are solely those of the authors and do not necessarily represent those of their affiliated organizations, or those of the publisher, the editors and the reviewers. Any product that may be evaluated in this article, or claim that may be made by its manufacturer, is not guaranteed or endorsed by the publisher.

## Footnotes

^1^ http://www.spirit-statement.org/
